# A Roentgen Study of 1000 Chests

**Published:** 1918-09

**Authors:** 


### *A Roentgen Study of 1000 Chests at Camp Devens, Mass.,* By E. L. Davis, Major, M. R. C. *Journal of the American Medical Association.* May 25, 1918.

The author presents his findings in more than 1000 chests. Some of the ideas expressed are practically new. The patients dealt with in this study are men between the ages of 21 and 30, officers and enlisted men of the army. All these men had passed the preliminary examination of local physicians. In practically every case reported in this paper the roentgen findings have been substantiated by the physical and clinical findings of examining surgeons.

He refers to instances in which the roentgen signs are decisive even though physical sounds are not found. Especially is this true in the cases of deep-seated infections, and incipient tuberculosis in a part of the lungs other than the seat of the main invaded area. The fact that many tuberculosis lesions are not found on physical examination, and that lesions are frequently larger in extent than determined by physical examination, would lead to the generalization that many cases of chronic fibroid tuberculosis give few or at least very indefinite signs in auscultation or percussion.

In examining roentgenograms of pneumonia patients made shortly after resolution, shadows are seen which appear strikingly similar to those found in pulmonary tuberculosis.

The roentgen signs of lobar pneumonia are: (1) marked accentuation of linear shadows from the hila to the apexes; (2) marked enlargement of the heart shadow; (3) typical shadow of the localized area of consolidation in the lungs; and (4) high diaphragm on the affected side.

The markings appear early in incipient pneumonia and disappear early in resolution. In the majority of cases the shadows indicative of vascular congestion disappear early in resolution. With vascular congestion in the upper lobes, the early enlargement of the heart which seems to be due to toxemia can usually be demonstrated and is a definite sign of beginning lobar pneumonia.

Consolidation in the lung shows as a definitely localized homogeneous patch varying in density from complete opacity to translucency.

In the presence of consolidation it is difficult to determine when fluid is present in sufficient amount to demand aspiration.

The author has found that broncho-pneumonia, generally supposed to be a bilateral disease, is more commonly unilateral, and that the heart is sometimes slightly enlarged and often not at all.

His summary is:

I. *Pulmonary Tuberculosis*:

(1) Pulmonary tuberculosis is demonstrated on the roentgenogram even in its earliest stage. It is difficult to describe concisely the appearance of shadows that will enable the roentgenologist definitely to interpret active and inactive tuberculosis. One can only describe what in general is seen in well defined cases.

(2) The lungs may be divided into three regions with regard to the occurrence of pulmonary tuberculosis: the upper region; the middle region in which tuberculosis is sometimes found; and the lower region in which tuberculosis is rarely found.

(3) Consultation with clinicians has led to the conclusion that many cases of chronic fibroid tuberculosis give very indefinite if any physical signs.

II. *Lobar Pneumonia*:

(1) Lobar pneumonia and broncho-pneumonia are easily distinguishable on the roentgenogram.

(2) Lobar pneumonia gives the following roentgen signs:

1. Vascular-lymphatic congestion in the upper lobes.
2. Enlarged heart.
3. Localized consolidation.
4. High diaphragm.

(3) Vascular-lymphatic thickening appears early and disappears early, though its occasional persistence may be confused with tuberculosis shadows.

(4) Pneumococcemia shows definitely on the roentgenogram where there are symptoms of pneumonia without consolidation, suggesting that the seriousness of the attack depends more on the toxemia than on the extent of pulmonary involvement.

(5) The heart is involved before definite signs of consolidation appear.

(6) Cardiac enlargement persists for some time after all signs of involvement of the lungs have disappeared.

(7) Consolidation in the affected side is suggestive but cannot be considered a constant sign.

III. *Broncho-pneumonia*:

(1) Broncho-pneumonia is more commonly unilateral than bilateral.

(2) The heart is usually not enlarged in broncho-pneumonia.

(3) Broncho-pneumonia is often overlooked on physical examination, as the symptoms may be indefinite and non-characteristic.

